# A Five-microRNA Signature as Prognostic Biomarker in Colorectal Cancer by Bioinformatics Analysis

**DOI:** 10.3389/fonc.2019.01207

**Published:** 2019-11-12

**Authors:** Guodong Yang, Yujiao Zhang, Jiyuan Yang

**Affiliations:** ^1^Department of Oncology, The First People's Hospital Affiliated to Yangtze University, Jingzhou, China; ^2^Respiratory Medicine, Huanggang Central Hospital Affiliated to Yangtze University, Huanggang, China

**Keywords:** microRNA, colorectal cancer, TCGA, prognosis, signature

## Abstract

Mounting evidence has demonstrated that a lot of miRNAs are overexpressed or downregulated in colorectal cancer (CRC) tissues and play a crucial role in tumorigenesis, invasion, and migration. The aim of our study was to screen new biomarkers related to CRC prognosis by bioinformatics analysis. By using the R language edgeR package for the differential analysis and standardization of miRNA expression profiles from The Cancer Genome Atlas (TCGA), 502 differentially expressed miRNAs (343 up-regulated, 159 down-regulated) were screened based on the cut-off criteria of *p* < 0.05 and |log2FC|>1, then all the patients (421) with differentially expressed miRNAs and complete survival time, status were then randomly divided into train group (212) and the test group (209). Eight miRNAs with *p* < 0.005 were revealed in univariate cox regression analysis of train group, then stepwise multivariate cox regression was applied for constituting a five-miRNA (hsa-miR-5091, hsa-miR-10b-3p, hsa-miR-9-5p, hsa-miR-187-3p, hsa-miR-32-5p) signature prognostic biomarkers with obviously different overall survival. Test group and entire group shown the same results utilizing the same prescient miRNA signature. The area under curve (AUC) of receiver operating characteristic (ROC) curve for predicting 5 years survival in train group, test group, and whole cohort were 0.79, 0.679, and 0.744, respectively, which demonstrated better predictive power of prognostic model. Furthermore, Univariate cox regression and multivariate cox regression considering other clinical factors displayed that the five-miRNA signature could serve as an independent prognostic factor. In order to predict the potential biological functions of five-miRNA signature, target genes of these five miRNAs were analyzed by Kyoto Encyclopedia of Genes and Genomes (KEGG) signaling pathway and Gene Ontology (GO) enrichment analysis. The top 10 hub genes (ESR1, ADCY9, MEF2C, NRXN1, ADCY5, FGF2, KITLG, GATA1, GRIA1, KAT2B) of target genes in protein protein interaction (PPI) network were screened by string database and Cytoscape 3.6.1 (plug-in cytoHubba). In addition, 19 of target genes were associated with survival prognosis. Taken together, the current study showed the model of five-miRNA signature could efficiently function as a novel and independent prognosis biomarker and therapeutic target for CRC patients.

## Introduction

CRC is a very common gastrointestinal tumor with high incidence and mortality. It was estimated that more than 1.8 million new colorectal cancer cases and 0.88 million deaths will occur in 2018, accounting for about 1 in 10 cancer about incidence and mortality (1). CRC patients usually show a survival rate of <5 years due to early metastasis. Although treatments (such as surgery, radiotherapy, chemotherapy, and targeted therapy) have been developed fleetly, high recurrence, and poor prognosis remain troubling issues (2). Although various biomarkers have been discovered and were associated with the occurrence, progression and prognosis of colorectal cancer to date (3), their reliability remains controversial. Consequently, it is urgent to screen new potential diagnostic and prognostic biomarkers or therapeutic targets for CRC.

MicroRNAs (miRNAs), a vital component of the non-coding RNA family, are approximately made up of 18–25 nucleotides, which almost function via binding 3′ untranslated regions(UTR)or 5′UTR of mRNA to suppress translation and promote mRNA cleavage (4). Along with the advances of human genome-sequencing technology, a great number of miRNAs have been abundantly discovered. Increasing evidence demonstrated that miRNAs regulated various oncogenesis processes including cellular proliferation, angiogenesis, differentiation, and apoptosis by binding oncogenes or tumor suppresser genes (5). Zhang et al. displayed miRNA-519b-3p functioned as a tumor suppressor miRNA to suppress colorectal cancer cell proliferation and invasion by regulating the umtck/wnt signaling pathway (6). Wang et al. exhibited that miRNA-496 accelerated epithelial-mesenchymal transition and migration of CRC via targeting RASSF6, which was involved in Wnt-pathway (7). Huang et al. demonstrated miR-506 inhibited cell proliferation, invasion, and migration of CRC via reducing NR4A1 expression (8). Studies on miRNA in colorectal cancer are far more than that, there are also some studies on miRNA as prognostic factors, including single, and multiple combinations. Although TCGA database has been used to construct the miRNA signature prognostic models for colon cancer (9, 10), there are still some shortcomings with no miRNAs matures, model validation, and risk assessment.

In the present study, we constructed, verified and assessed a novel five-miRNA signature that predicted effectively over survival of CRC patients derived from TCGA database. Functional enrichment analysis revealed potential biological functions and signal pathways of five-miRNA signature associated with cancer, which enhances our understanding to molecular mechanisms of model in CRC.

## Materials and Methods

### Data Download and Processing

The miRNA expression information [Case (455): Primary Site (Colon and Rectum), Program (TCGA),Project (TCGA-COAD and TCGA-READ), Disease Type (Adenomas and Adenocarcinomas); Files(473): Data Category (Transcriptome Profiling), Data Type (Isoform Expression Quantification)], mRNA expression information [Case (472): Primary Site (Colon and Rectum), Program (TCGA), Project (TCGA-COAD and TCGA-READ), Disease Type (Adenomas and Adenocarcinomas); Files (530): Data Category (Transcriptome Profiling), Data Type (Gene Expression Quantification)] and their related clinical information (476) (Data Category: Clinical, Data Format: BCR XML) (Table 1) of all colorectal cancer samples were downloaded from The Cancer Genome Atlas (TCGA) official website (https://cancergenome.nih.gov/) on July 3, 2019, the former of which contained 464 tumor samples and 9 normal samples, the latter included 488 tumor samples and 42 normal samples. The Fasta format sequences of all mature miRNA sequences (mature.fa) were downloaded from the miRBase website (http://www.mirbase.org/). We combined these two sets of data in the Perl language to obtain expression profile information for each mature miRNA.

**Table 1:** Summary of patient cohort information.

**Variables**	**Case**	**Percentage**
**GENDER**		
Male	256	53.78%
Female	220	46.22%
**AGE (YEARS)**		
Range	31–90	
Median	68	14.29
**RACE**		
ASIAN	9	1.89%
BLACK	52	10.92%
WHITE	219	46.01%
Unknown	196	41.18%
**CLINICAL STAGE**		
Stage I	85	17.86%
Stage II	180	37.82%
Stage III	126	26.47%
Stage IV	70	14.71%
Unknown	15	3.15%
**T STAGE**		
T1+Tis	15	3.15%
T2	86	18.07%
T3	324	68.07%
T4	51	10.71%
**LYMPH NODE STATUS**		
N0	282	59.24%
N1	114	23.95%
N2	80	16.81%
Nx	1	0.21%
**METASTATIC**		
M0	355	74.58%
M1	69	14.50%
Mx	45	9.45%
Unknown	7	1.47%
**CANCER TYPE**		
COAD	385	80.88%
READ	91	19.12%

### Identification of Differentially Expressed miRNAs, mRNA, and Their Combination With Patient Survival Data

We used R language 3.6.1 version edgeR package to compare the miRNA and mRNA expression of tumor group with normal group and normalize the expression profile of miRNAs and mRNA, whose mean value was >1, the screening criteria were corrected *p* value (FDR) < 0.05 and |log2FC|>1 (11). We selected the clinical information of patients with survival time ≥30 days and combined it with differentially expressed and standardized miRNA and mRNA expression profiles.

### Grouping of Samples and Construction, Validation, and Evaluation of Prognostic Models

We used the R language 3.6.1 version “caret” package to randomly divide the samples with complete survival information and differentially expressed miRNA expression profiles into two groups (train group and test group), and performed univariate Cox regression analysis of miRNAs for the train group.

In order to reduce the number of miRNAs with similar expression, miRNAs with *p* value < 0.005 were subjected to a stepwise multivariate Cox regression to construct the prognostic model. In the multivariate Cox regression analysis, we took advantage of the function of “Coxph” and “direction = both” in R language survival package (12). Then, the risk score of a prognostic miRNA signature comprising multiple miRNAs was established based on the summation of the product of each miRNA and its coefficient. Furthermore, we tested the Proportional Hazards Assumption in Cox model. This model was used to evaluate the survival prognosis of each patients in train group, test group, entire group using Kaplan-Meier curve, and log-rank test according to median value grouping of risk score, namely high risk group, and low risk group. The predictive power of the miRNA signature was assessed by calculating AUC of 3 years dependent ROC curve using “survivalROC” package (13).

### Independent Prognostic Ability of the miRNA Signature Including Other Clinical Variables

The relationship between the prognostic miRNA signature and patients' overall survival was analyzed in the train group by univariate Cox regression, as well as clinical variables (including age, gender, and clinical stage, lymph nodes, distant metastasis). Variables with *p* value < 0.05 in univariate Cox regression were further used for multivariate Cox regression analysis to determine whether they could function as independent prognostic factors. In order to compare the predictive power of this risk model compared to other clinical characteristics, we have drawn ROC curves for this model risk score and clinical characteristics. In addition, we tested the correlation of each miRNA to clinical features by using the SPSS 21.0 chi-square test, with a *p*-value of < 0.05 being considered meaningfully.

### Target Genes Prediction of miRNA Signature and Their Potential Functions

We downloaded the miRNA prediction database from three miRNA target gene prediction websites including miRTarBase (http://mirtarbase.mbc.nctu.edu.tw/), targetScan (http://www.targetscan.org) and miRDB (http://www.mirdb.org/), and used the Perl language to find the target genes of miRNA signature which are covered in at least 2 databases, meanwhile, utilizing the Venn diagram, and Cytoscape 3.6.1 to map the relationship between the miRNA and these target genes. To clarify whether the target genes of these miRNAs are likely to participate in the progression of colorectal cancer, we taken the intersection of these target genes and differentially expressed genes in colorectal cancer. All of these intersection genes obtained were analyzed by Kyoto Encyclopedia of Genes and Genomes (KEGG) signaling pathway and Gene Ontology (GO) enrichment analysis through the R language “clusterProfiler” package (14) and the “org.Hs.eg.db” package, The *p* adjust < 0.05 and *q* value < 0.05 was set as the cut-off criteria.

### Screening of Hub Genes and Survival Related Gene

The PPI network of the STRING database (https://string-db.org/) (15) was applied to unearth the relationship between the target genes, the parameter of settings the medium confidence is 0.400. Then, the network relationship file was downloaded and the top 10 hub genes were identified in accordance with Cytoscape 3.6.1 and its plug-in (degrees ranking of cytoHubba). Meanwhile, The Kaplan-Meier method was used to check whether the intersection gene is related to over survival, log rank test < 0.05.

### Statistical Analysis

All statistical analyses are based on R language 3.6.1 version and attached packages.

## Results

### Identification of Differentially Expressed miRNAs and mRNAs

**Figure 1:**
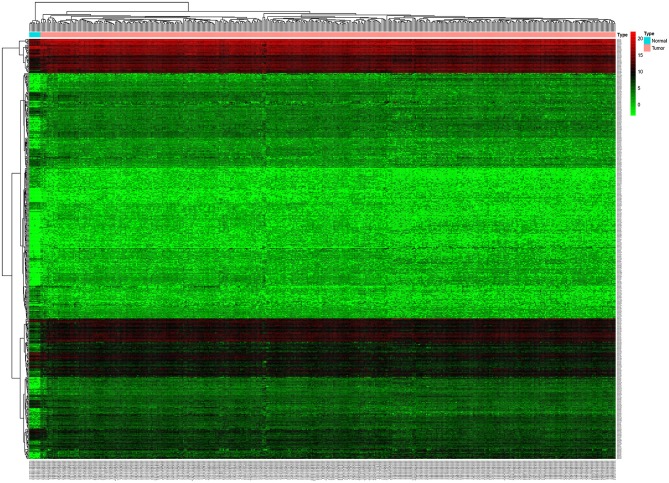
Unsupervised hierarchical clustering heatmap based on the differentially expressed miRNAs between 464 colorectal cancer tissues and 9 normal tissues.

Based on this screening criteria, miRNA mature expression profiles between 464 tumor samples and 9 normal samples showed 502 differentially expressed miRNAs (DEmiRNAs), of which 343 were up-regulated and 159 were down-regulated (Figure 1). mRNA expression profiles between 488 tumor samples and 42 normal samples showed 5,540 differentially expressed mRNAs (DEmRNAs), of which 2992 were up-regulated and 2,548 were down-regulated, displayed in Supplemental Table 1.

### Construction of the Predictive Five-miRNA Signature

**Figure 2:**
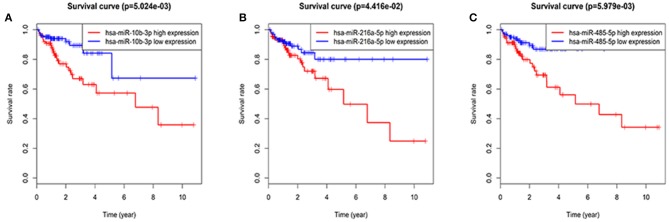
Three miRNAs associated with overall survival in CRC patients using Kaplan–Meier curves and log-rank tests. The patients were stratified into high and low expression groups according to the median expression of each miRNA. **(A)** hsa-miR-10b-3p. **(B)** hsa-miR-216a-5p. **(C)** hsa-miR-485-5p.

The entire group (*N* = 421) with miRNA mature expression profiles was randomly divided into train group (*N* = 212) (Supplemental Table 2) and test group (*N* = 209) (Supplemental Table 3). The univariate Cox regression analysis displayed that a total of thirty-two miRNAs were found to be associated with patients' overall survival (*p* value < 0.05) in the train group. For the reliability of the model, eight miRNAs (*p* value < 0.005) were selected for further analysis (Table 2). Kaplan-Meier method pointed out hsa-miR-10b-3p, hsa-miR-216a-5p, and hsa-miR-485-5p of eight miRNAs were associated with patients' overall survival (*p* value < 0.05; Figure 2), however, the high expression of hsa-miR-485-5p with poor prognosis and the fact that hsa-miR-485-5p exhibited low expression in tumors is contradictory. Therefore, the remaining seven miRNAs were targeted for further analysis.

**Table 2:** Univariate and multivariate Cox regression of differentially expressed miRNAs.

	**Univariate Cox regression**				**Multivariate Cox regression**			
**id**	**HR**	**HR.95L**	**HR.95H**	***P* value**	**Co ef**	**HR**	**HR.95L**	**HR.95H**	***P* value**
hsa-miR-485-5p	1.292	1.124	1.485	0.000					
hsa-miR-216a-5p	1.069	1.031	1.109	0.000					
hsa-miR-187-3p	1.044	1.019	1.069	0.000	0.031	1.031	1.001	1.062	0.041
hsa-miR-10b-3p	1.016	1.006	1.027	0.003	0.011	1.011	0.999	1.023	0.067
hsa-miR-32-5p	1.007	1.003	1.012	0.003	0.008	1.008	1.003	1.013	0.003
hsa-miR-9-5p	1.000	1.000	1.000	0.003	0.000	1.000	1.000	1.000	0.008
hsa-miR-5091	1.194	1.059	1.346	0.004	0.177	1.194	1.045	1.363	0.009
hsa-miR-5683	1.004	1.001	1.006	0.005					

Based on the previous research, Five (hsa-miR-5091, hsa-miR-10b-3p, hsa-miR-9-5p, hsa-miR-187-3p, hsa-miR-32-5p) of the seven candidate miRNAs therein were finally screened out (Table 2) by stepwise multivariate Cox regression analysis, then a predictive miRNA signature model was established on the summation of the product of each miRNA and its coefficient in multivariate Cox regression as follows: miRNA signature risk score = (0.1769 × expression of hsa-miR-5091) + (0.0110 × expression of hsa-miR-10b-3p) + (0.0001 × expression of hsa-miR-9-5p) + (0.0305 × expression of hsa-miR-187-3p) + (0.0076 × expression of hsa-miR-32-5p). In addition, the results testing the Proportional Hazards Assumption in Cox model demonstrated that all the *P* values are higher than 0.05, which means that they meet the PH test (Supplemental Table 4).

### Prediction of the Five-miRNA Signature for Over Survival in the Train Group, Test Group, and Entire Group

**Figure 3:**
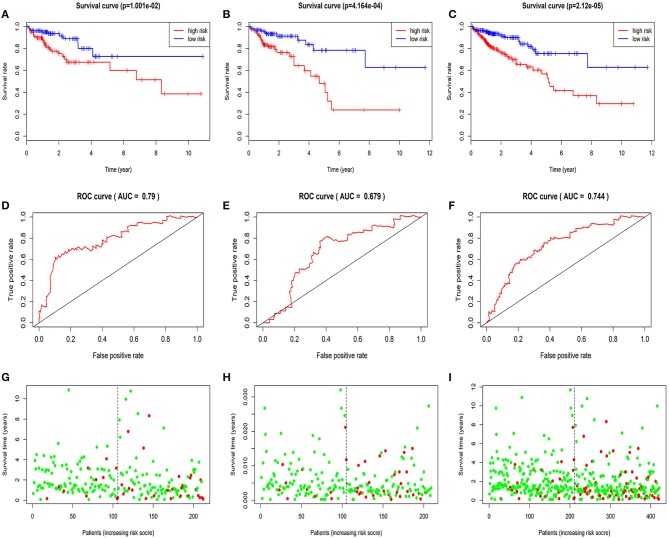
Validation and evaluation of the predictive five-miRNA signature. Kaplan-Meier curves in the train group **(A)**, test group **(B)**, entire group **(C)**; The AUC of three years dependent curve in the train group **(D)**, test group **(E)**, entire group **(F)**, Survival status in high and low risk patients for train group **(G)**, test group **(H)**, entire group **(I)**, red dots represent death, green dots represent alive.

Based on median value grouping of risk score. Kaplan-Meier curves shown high risk group had an obviously poorer overall survival compared to low risk group in the train group (*p* = 1.001E-02), test group (*p* = 4.164E-04) and entire group (*p* = 2.12E-05; Figures 3A–C). The train group shown overall survival of 5 years for patients with high and low risk group were 60.0 and 72.8%, respectively. The test group demonstrated that overall survival of 5 years for patients with high and low risk group were 39.9 and 62.7%, respectively. The entire group displayed that overall survival of 5 years for patients with high and low risk group were 53.0 and 62.8%, respectively.

### Evaluation of the Five-miRNA Signature for Over Survival in the Train Group, Test Group, and Entire Group

The AUC of 3 years dependent ROC for the five-miRNA signature achieved 0.790, 0.679, 0.744, respectively, in the train group, test group and entire group (Figures 3D–F), which demonstrated the better performance of model in predicting CRC patient survival risk. In addition, in the three groups, the patients with high risk score had higher mortality rates than low (Figures 3G–I).

### Independence of the Five-miRNA Signature Considering Other Clinical Factors

**Figure 4:**
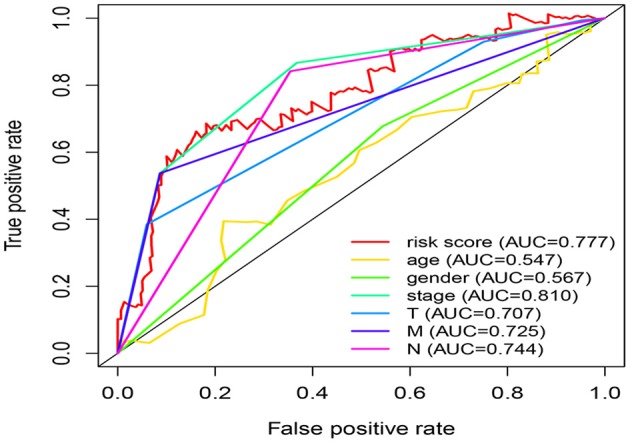
Comparison of risk score and clinical features in predicting the accuracy of patients' survival prognosis.

Univariate Cox regression analysis exhibited that the five-miRNA signature was evidently associated with patients' overall survival (hazard ratio HR = 1.286, confidence interval 95% CI = 1.164–1.420, *p* = 6.719E-07; Table 3). Multivariate Cox regression analysis pointed out that the five-miRNA signature remained independent with overall survival considering other conventional clinical factors (HR = 1.326, 95% CI = 1.168–1.505, *p* = 1.23E-05), such as clinical stage, T stage, Lymph-node status, distant metastasis, which makes it possible to be a prognostic marker for CRC in the future. Meanwhile, distant metastasis was also found to be an independent prognostic factor (HR = 2.976, 95% CI = 1.285–6.891, *p* = 0.01). The ROC curves for this model risk score and clinical characteristics demonstrated that risk score (0.777), clinical stage (0.810), T stage (0.707), Lymph-node status (0.725), and distant metastasis (0.744) had a high predictive ability (Figure 4). In addition, the results about the correlation of each miRNA to clinical features demonstrated hsa-miR-10b-3p was associated with T stage (*p* = 0.011), hsa-miR-9-5p was associated with age (*p* = 0.032), and clinical stage (*p* = 0.049), hsa-mir-3189 was associated with Metastasis (*p* = 0.002) and clinical stage (*p* = 0.042; Table 4), which further suggested that these miRNAs do have a close relationship with some clinical features.

**Table 3:** Univariate and multivariate Cox regression of clinical features.

**Clinical features**	**Univariate Cox regression**				**Multivariate Cox regression**			
	**HR**	**HR.95L**	**HR.95H**	***P* value**	**HR**	**HR.95L**	**HR.95H**	***P* value**
Age (continuous variable)	1.017	0.986	1.050	0.290				
Gender (male vs. female)	1.210	0.594	2.467	0.600				
Clinical stage (III+IV vs. I+II)	7.872	3.010	20.588	0.000	4.902	0.472	50.935	0.183
T stage (T3+4 vs. T1+2)	6.694	0.910	49.250	0.062				
M (M1 VS M0)	7.920	3.816	16.440	0.000	2.977	1.286	6.892	0.011
N (N1+2 vs. N0)	6.585	2.693	16.102	0.000	0.996	0.130	7.666	0.997
Five-miRNA signature	1.286	1.165	1.420	0.000	1.326	1.168	1.505	0.000

**Table 4:** The correlation of each miRNA to clinical features.

**Variables**	**Numbers**	**hsa-miR-5091**		**χ^2^ test *P* value**	**hsa-miR-10b-3p**		**χ^2^ test *P* value**	**hsa-miR-9-5p**		**χ^2^ test P value**	**hsa-miR-187-3p**		**χ^2^ test *P* value**	**hsa-mir-3189**		**χ^2^ test *P* value**
		**Low Expression**	**High Expression**		**Low Expression**	**High Expression**		**Low Expression**	**High Expression**		**Low Expression**	**High Expression**		**Low Expression**	**High Expression**	
**GENDER**																
Female	81	46	35	0.12	43	38	0.425	44	37	0.334	40	41	0.834	41	40	0.4
Male	108	49	59		51	57		51	57		55	53		48	60	
**AGE AT DIAGNOSIS**																
>60	136	69	67	0.836	66	70	0.595	75	61	0.032	72	64	0.238	59	77	0.102
≤ 60	53	26	27		28	25		20	33		23	30		30	23	
**T STAGE**																
T1+2	40	23	17	0.303	27	13	0.011	21	19	0.75	25	15	0.081	20	20	0.678
T3+4	149	72	77		67	82		74	75		70	79		69	80	
**METASTASIS**																
M0	155	78	77	0.973	79	76	0.469	81	74	0.242	83	72	0.054	81	74	0.002
M1	34	17	17		15	19		14	20		12	22		8	26	
**LYMPH NODE STATUS**																
N0	103	53	60	0.72	54	49	0.418	58	45	0.069	55	48	0.346	54	49	0.108
N1-2	86	42	44		40	46		37	49		40	46		35	51	
STAGE																
I+II	100	50	50	0.939	52	48	0.509	57	43	0.049	52	48	0.613	54	46	0.044
III+IV	89	45	44		42	47		38	51		43	46		35	54	

### Prediction of Target Genes for the Five miRNAs

**Figure 5:**
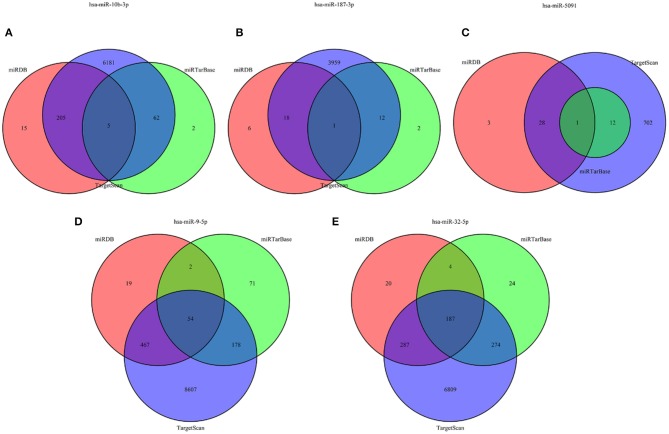
Venn diagram of target genes for five miRNAs. **(A)** hsa-miR-10b-3p, **(B)** hsa-miR-187-3p, **(C)** hsa-miR-5091, **(D)** hsa-miR-9-5p, **(E)** hsa-miR-32-5p.

**Figure 6:**
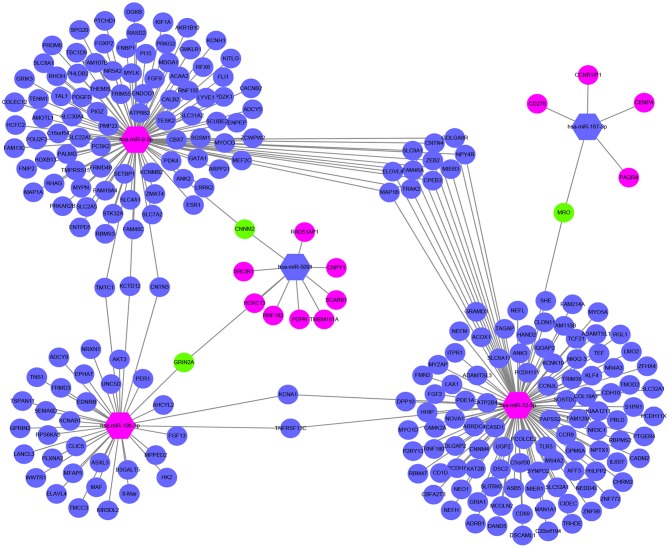
Sub network map of miRNA regulating mRNA. The hexagon represents miRNA. The circle stands for mRNA. Red means upregulated, blue means downregulated, and green means both.

The target genes regulated by the five miRNAs, were predicted in at least 2 databases. To further enhance the reliability of the bioinformatic analysis, the overlapping target genes were identified. The results indicated that 41, 272, 701, 31, and 752 overlapping genes were identified for hsa-miR-5091, hsa-miR-10b-3p, hsa-miR-9-5p, hsa-miR-187-3p, hsa-miR-32-5p, respectively, by the three databases above, which were shown using Venn diagram (Figure 5) and network map of miRNA-target genes (Supplemental Figure 1). A total of 1,672 target genes was predicted for the five miRNAs. To clarify whether the target genes of these miRNAs are likely to participate in the progression of CRC, the above obtained 5540 DEmRNAs (up-regulated 2992, down-regulated 2548) was used for analysis. The intersection of target mRNAs for down-regulated miRNAs (hsa-miR-5091, hsa-miR-187-3p) and upregulated mRNAs, and target mRNAs for upregulated miRNAs (hsa-miR-32-5p, hsa-miR-10b-3p, hsa-miR-9-5p) and downregulated mRNAs were taken. The results were performed on a total of 246 genes including 12 up-regulated genes, 234 down-regulated genes, respectively (Supplemental Figure 2). The sub network between the five miRNAs and their 246 target genes was shown in Figure 6.

### Functional Enrichment Analysis of Target Genes Associated CRC

**Figure 7:**
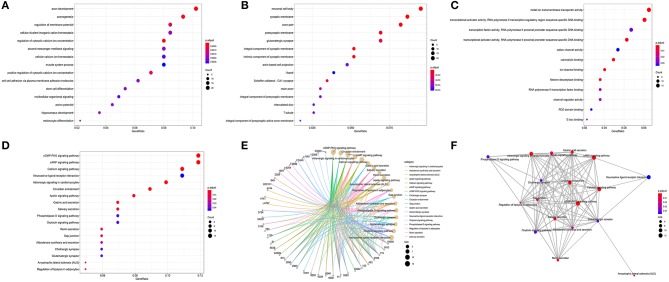
Functional enrichment analysis of target genes associated CRC. **(A)** BP, **(B)** CC, **(C)** MF, **(D)** dotplot of KEGG signal pathway shown the counts of genes, **(E)** cnetplot of KEGG signal pathway shown the “pathway-gene” network, **(F)** emapplot of KEGG signal pathway shown the “pathway-pathway” network.

The results of GO annotation about the target genes associated CRC are 234 (Supplemental Table 5). The top fifteen terms from the GO results: biological process (BP), cellular component (CC), and molecular function (MF) were demonstrated in dotplot (Figures 7A–C). In the three categories, BP analysis mostly include axon development, axonogenesis, and stem cell differentiation, CC analysis was mainly contained synaptic membrane, postsynaptic membrane and neuronal cell body, MF analysis mainly contained metal ion transmembrane transporter activity, transcriptional activator activity and DNA binding, ion channel binding. The results of KEGG pathways about the target genes associated CRC are 18 (Table 5), of which counts > 10 were mainly enriched in the cGMP-PKG signaling pathway, cAMP signaling pathway, Calcium signaling pathway, Neuroactive ligand-receptor interaction In addition, to provide a readable graphic representation of the complex relationship between target genes and relative KEGG pathway, the “pathway-gene network” and “pathway-pathway network” was also shown in Figures 7D–F.

**Table 5:** KEGG pathways of target genes associated CRC.

**ID**	**Description**	**P Adjust**	**Q value**	**Count**	**Gene ID**
hsa04022	cGMP-PKG signaling pathway	0.00028	0.000212	12	AKT3/EDNRB/ADCY9/ITPR1/ATP2B4/ADRB1/KCNMB2/PRKG2/ADCY5/MYLK/SLC8A1/MEF2C
hsa04713	Circadian entrainment	0.000397	0.0003	9	RPS6KA5/PER1/GRIN2A/ADCY9/ITPR1/GRIA1/CAMK2A/PRKG2/ADCY5
hsa04024	cAMP signaling pathway	0.001032	0.000782	12	AKT3/GRIN2A/ADCY9/ATP2B4/HHIP/SLC9A1/GRIA1/CAMK2A/ACOX1/ADRB1/CHRM2/ADCY5
hsa04261	Adrenergic signaling in cardiomyocytes	0.001032	0.000782	10	RPS6KA5/AKT3/ADCY9/ATP2B4/SLC9A1/CAMK2A/ADRB1/ADCY5/SLC8A1/CACNB2
hsa04020	Calcium signaling pathway	0.001443	0.001093	11	GRIN2A/EDNRB/ADCY9/ITPR1/ATP2B4/CAMK2A/ADRB1/PDE1A/CHRM2/MYLK/SLC8A1
hsa04971	Gastric acid secretion	0.001615	0.001223	7	ADCY9/ITPR1/SLC9A1/KCNK10/CAMK2A/ADCY5/MYLK
hsa04970	Salivary secretion	0.004453	0.003373	7	ADCY9/ITPR1/ATP2B4/SLC9A1/ADRB1/PRKG2/ADCY5
hsa04924	Renin secretion	0.006429	0.00487	6	PTGER4/ITPR1/ADRB1/PDE1A/PRKG2/ADCY5
hsa04371	Apelin signaling pathway	0.008483	0.006426	8	AKT3/ADCY9/ITPR1/SLC9A1/ADCY5/MYLK/SLC8A1/MEF2C
hsa05014	Amyotrophic lateral sclerosis (ALS)	0.009933	0.007525	5	GRIN2A/NEFL/NEFM/NEFH/GRIA1
hsa04923	Regulation of lipolysis in adipocytes	0.012842	0.009728	5	AKT3/ADCY9/ADRB1/PRKG2/ADCY5
hsa04540	Gap junction	0.015926	0.012064	6	ADCY9/ITPR1/ADRB1/PDGFD/PRKG2/ADCY5
hsa04925	Aldosterone synthesis and secretion	0.025762	0.019515	6	ADCY9/ITPR1/ATP2B4/CAMK2A/ADCY5/SCARB1
hsa04072	Phospholipase D signaling pathway	0.04312	0.032663	7	AKT3/ADCY9/MS4A2/DGKB/PDGFD/ADCY5/KITLG
hsa04725	Cholinergic synapse	0.04312	0.032663	6	AKT3/ADCY9/ITPR1/CAMK2A/CHRM2/ADCY5
hsa04724	Glutamatergic synapse	0.04312	0.032663	6	GRIN2A/ADCY9/ITPR1/GRIA1/ADCY5/GRIK3
hsa04921	Oxytocin signaling pathway	0.04312	0.032663	7	ADCY9/ITPR1/CAMK2A/ADCY5/MYLK/MEF2C/CACNB2
hsa04080	Neuroactive ligand-receptor interaction	0.046345	0.035106	11	GRIN2A/EDNRB/PTGER4/NPY4R/GRIA1/S1PR1/NR3C1/P2RY13/ADRB1/CHRM2/GRIK3

### Hub Genes of PPI Network and Survival Related Target Genes

**Figure 8:**
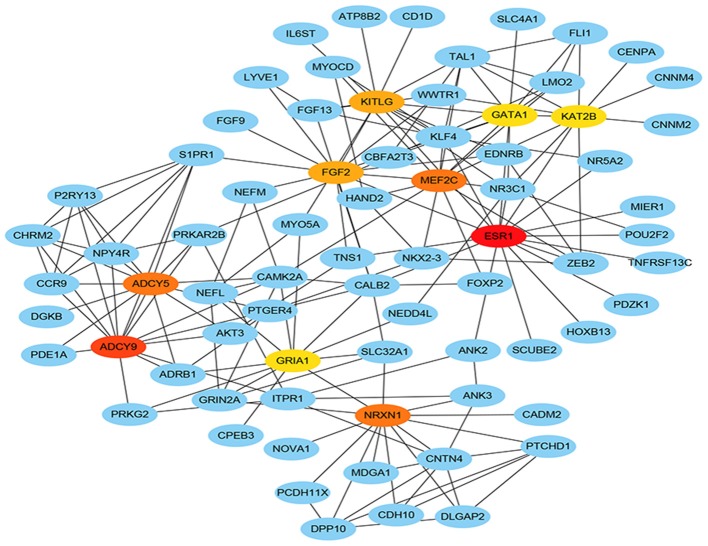
Hub genes of PPI network. The darker the color, the bigger the degrees.

**Figure 9:**
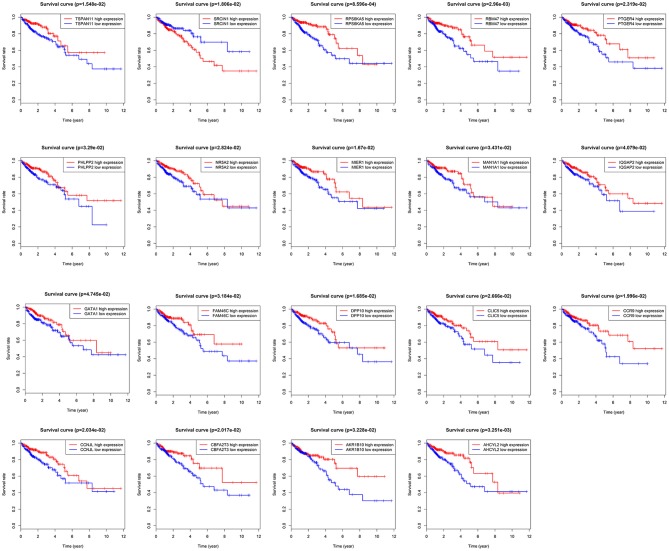
Target genes associated with over survival.

Total of 244 of the 246 target genes were filtered into the target genes PPI network complex, containing 178 nodes and 326 edges, 10 hub gene (ESR1, ADCY9, MEF2C, NRXN1, ADCY5, FGF2, KITLG, GATA1, GRIA1, KAT2B) were screened according to Cytoscape 3.6.1 and its plug-in (degree ranking of cytoHubba) (Figure 8 and Table 6). In addition, Kaplan-Meier method showed that the expression of 18 of the 246 genes (AHCYL2, AKR1B10, CBFA2T3, CCNJL, CCR9, CLIC5, DPP10, FAM46C, GATA1, IQGAP2, MAN1A1, MIER1, NR5A2, PHLPP2, PTGER4, RBM47, RPS6KA5, TSPAN11) were positively associated with survival prognosis, however, the high expression of SRCIN1 shown a poorer over survival (Figure 9).

**Table 6:** Identification of hub genes by cytoHubba.

**Node_name**	**MCC**	**DMNC**	**MNC**	**Degree**	**EPC**	**Bottle Neck**	**Ec Centricity**	**Closeness**	**Radiality**	**Betweenness**	**Stress**	**Clustering Coefficient**
ESR1	40	0.238	11	17	57.77	46	0.101	67.513	10.95	7030.605	17450	0.103
ADCY9	742	0.282	14	14	56.906	4	0.113	59.613	10.577	1678.562	6756	0.275
MEF2C	38	0.255	11	13	57.288	14	0.113	61.663	10.735	2113.807	7622	0.192
NRXN1	44	0.321	8	13	44.099	20	0.113	54.98	10.317	3224.563	11316	0.179
ADCY5	739	0.337	12	13	56.085	13	0.113	56.846	10.453	1075.731	4490	0.295
FGF2	19	0.256	7	12	57.027	15	0.101	63.513	10.826	2905.838	9030	0.106
KITLG	32	0.321	8	12	56.272	9	0.09	60.182	10.639	1839.913	5646	0.167
GATA1	67	0.419	10	11	56.982	8	0.101	58.69	10.566	1203.977	3882	0.382
GRIA1	22	0.329	7	11	54.01	26	0.113	61.78	10.803	3086.888	10038	0.164
KAT2B	32	0.402	7	11	53.712	9	0.101	57.856	10.498	1381.704	4194	0.2

## Discussion

Colorectal cancer is a highly malignant tumor, which is particularly prone to liver and lung metastasis, seriously affecting the survival prognosis of patients (16). Therefore, finding a prognostic marker with high specificity and sensitivity is becoming more and more urgent for patients. Extensive evidence displayed miRNAs can regulate the expression of abundant genes, playing critical roles in many biological processes of human malignant tumor (17). Especially, recent studies have revealed that distinct miRNA-expression profiles seriously affected the development and progression of CRC (18, 19). At present, several miRNAs are known to be used as potential prognostic indictors in various cancers, including miR-191 (20), miR-1908 (21), miR-200c (22), and miR-217 (23). However, overwhelming studies manifested that multiple miRNA signature have bigger advantages than single miRNA on the hand of statistically robust analysis. Thence before our study, there have been a lot of prognostic markers based on multiple miRNA signature in tumors (24–26), especially colorectal cancer (9, 10, 27). There are many differences between our research and previous studies yet, such as research methods, sample size, and most importantly, we use miRNA matures and sample groupings to validate the model.

In the current study, we download mature miRNA expression profiles and corresponding patients' clinical information of CRC from TCGA database. By using the R language edgeR package for the differential analysis, 502 DEmiRNAs were obtained. All the patients were randomly divided into train group and test group, then a five-miRNA signature model (hsa-miR-5091, hsa-miR-10b-3p, hsa-miR-9-5p, hsa-miR-187-3p, hsa-miR-32-5p) was constructed by univariate Cox regression and stepwise multivariate Cox regression in train group. Meanwhile, a five-miRNA signature was validated in test group and entire group. Based on median value grouping of risk score. Kaplan-Meier curves shown high risk group had an obviously poorer overall survival compared to low risk group in the three group. Evaluation of the five-miRNA signature for over survival in the three group by ROC curve displayed better predictive power. Univariate Cox regression and multivariate Cox regression analysis also pointed out that the five-miRNA signature remained independent with overall survival considering other conventional clinical factors for CRC patients. Most of these five miRNAs have been reported to participate in the research progress of various tumors. Lu et al. demonstrated that the expression level of mir-10b-3p was obviously upregulated in tumor and serum samples of esophageal cancer (ESCC) patients. The expression level of mir-10b-3p is not only correlated with lymph node metastasis and clinical staging, but also serves as an independent prognostic biomarker for overall survival of ESCC patients. Augmented expression of mir-10b-3p stimulates cell proliferation, invasion, and migration through directly combining the FOXO3 3'UTR in ESCC (28). Chen et al. shown that miR-9-5p expression was upregulated in prostate cancer cells, functioned as oncogene role in the proliferation, migration, invasion, and epithelial-mesenchymal transition (EMT) of prostate cancer cells by binding StarD13 (29). Dou et al. demonstrated that miR-187-3p was lowly expressed in hepatic carcinoma (HCC) tissues and cell lines, and was not only correlated with clinical stage and metastasis of HCC, but also accelerated effects of hypoxia on EMT of HCC cells. Furthermore, miR-187-3p suppressed EMT process in HCC via regulating S100A4 (30). Fu et al. reported that miR-32-5p was markedly upregulated in the HCC multidrug-resistant cell line (Bel/5-FU). Overexpression of miR-32-5p demonstrated a worse prognosis, miR-32-5p regulated the PI3K/Akt pathway via inhabiting PTEN and leaded to multidrug resistance by exosomes, then advanced epithelial-mesenchymal transition (EMT) and angiogenesis (31). However, the current research mechanism of hsa-miR-5091 in tumors has not been reported yet, so more experiments in the future need to be carried out to hsa-miR-5091, especially in CRC.

To further understand the regulatory mechanism of the five-miRNA signature in colorectal cancer, the target genes of five miRNAs in the model were predicted by three target gene prediction databases. At the same time, based on the study of colorectal cancer, we obtained the intersection of the target genes of these miRNAs and the differentially expressed genes from the TCGA database, and performed functional enrichment analysis on these intersection genes. The GO annotation of the target genes was mainly associated with axon development, axonogenesis and stem cell differentiation, synaptic membrane, postsynaptic membrane, and neuronal cell body, metal ion transmembrane transporter activity, transcriptional activator activity and DNA binding, ion channel binding. The signal pathways of the target genes mainly enriched in the cGMP-PKG signaling pathway, cAMP signaling pathway, Calcium signaling pathway, Neuroactive ligand-receptor interaction. Ren et al. illuminated that the cGMP/PKG signaling pathway played an essential role on proliferation and survival of human renal carcinoma cells (32). Park et al. displayed that the cAMP signaling pathway regulated by the Epac-Rap1-Akt pathway caused suppression of JNK-dependent HDAC8 degradation, which augments cisplatin-induced apoptosis by inhabiting TIPRL expression in lung cancer cells (33). Monteith GR reviewed that calcium signaling pathway not only played key role on proliferation, invasion and sensitivity to cell death, but also in the establishment and maintenance of multidrug resistance and the tumor microenvironment (34). These signaling pathways show their effects on tumors to varying degrees, and these three signaling pathways are only the tip of the iceberg of the target gene involved in signaling pathway, which prompts that our constructed miRNA prognosis model may be involved in the regulation of tumor signaling pathways.

In order to find key nodes of the miRNA signature model regulating colorectal cancer 10 hub genes (ESR1, ADCY9, MEF2C, NRXN1, ADCY5, FGF2, KITLG, GATA1, GRIA1, KAT2B) were screened according to Cytoscape 3.6.1 and its plug-in (degree ranking of cytoHubba). In addition, the Kaplan-Meier method showed that the expression of 18 genes (AHCYL2, AKR1B10, CBFA2T3, CCNJL, CCR9, CLIC5, DPP10, FAM46C, GATA1, IQGAP2, MAN1A1, MIER1, NR5A2, PHLPP2, PTGER4, RBM47, RPS6KA5, TSPAN11) were positively associated with survival prognosis, however the high expression of SRCIN1 shown a poorer over survival. Surprisingly, GATA1 (GATA binding protein 1) is not only a key gene in the PPI network, but also related to over survival of patients, which encodes s a protein which belongs to the GATA family of transcription factors and promoted erythroid development via adjusting the switch of fetal hemoglobin to adult hemoglobin. Wang et al. pointed out that decreased of GATA-1 was to the benefit of high expression of IRF-3 in lung adenocarcinoma cells by binding with a specific domain of IRF-3 promoter, consequently, alternating the immunomodulatory function in tumorigenesis (35). Thus, the miRNA signature may affect the survival prognosis of colorectal cancer patients and the colorectal cancer progression through regulating GATA1.

## Conclusion

In summary, our study not only constructed a new predictive model of miRNA signature prognosis through miRNA mature expression profiling, but also by grouping to verify and evaluating the predictive ability of the model, the most important thing is that it can be used as an independent prognostic factors in CRC. In addition, the potential function is inferred by predicting the target genes of the model, which enhance our comprehension to tumorigenesis and progression of CRC. However, this is just a study based on the TCGA database using bioinformatics. We hope that there will be other databases and a large number of experiments to verify the feasibility of this prognostic model in the future and provide a reliable predictor and therapeutic target for CRC patients.

## Data Availability Statement

The raw data supporting the conclusions of this manuscript will be made available by the authors, without undue reservation, to any qualified researcher.

## Author Contributions

YZ downloaded the miRNA and mRNA expression information. GY constructed miRNA signature model and performed the statistical analysis using R language software, and wrote the first draft of the manuscript. JY contributed conception and design of the study and checked the manuscript.

### Conflict of Interest

The authors declare that the research was conducted in the absence of any commercial or financial relationships that could be construed as a potential conflict of interest.

## Supplementary Material

Supplemental Figure 1 The network map between miRNAs and target genes. The hexagon represents miRNA, the circle stands for mRNA. Red means upregulated, blue means downregulated.

Supplemental Figure 2 The intersection of target mRNAs for miRNA and differentially expressed mRNAs.

Supplemental Table 1 Differentially expressed mRNAs between colorectal cancer samples and normal samples.

Supplemental Table 2 GO annotation of the target genes.

Supplemental Table 3 Survival information of differentially expressed miRNA train group.

Supplemental Table 4 Proportional Hazards Assumption in Cox model.

Supplemental Table 5 GO annotation of the target genes.
