# Supplementary figures and images for: A Five-microRNA Signature as Prognostic Biomarker in Colorectal Cancer by Bioinformatics Analysis
Source: Front Oncol. 2019 Nov 12;9:1207. doi: 10.3389/fonc.2019.01207 (PMC6863365; doi:10.3389/fonc.2019.01207)

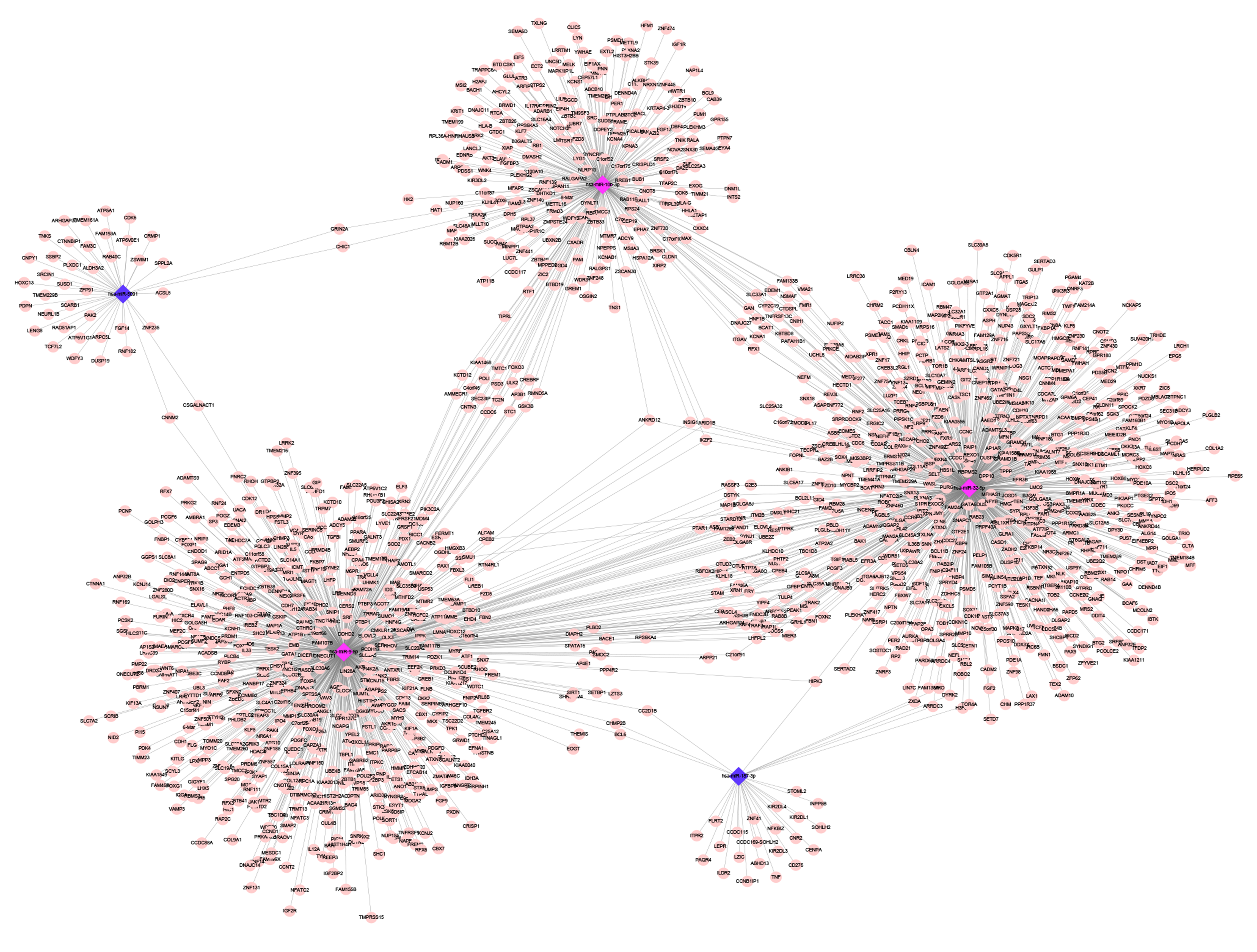

Supplement: Supplemental Figure 1 — The network map between miRNAs and target genes. The hexagon represents miRNA, the circle stands for mRNA. Red means upregulated, blue means downregulated. [file Image_1.TIFF]

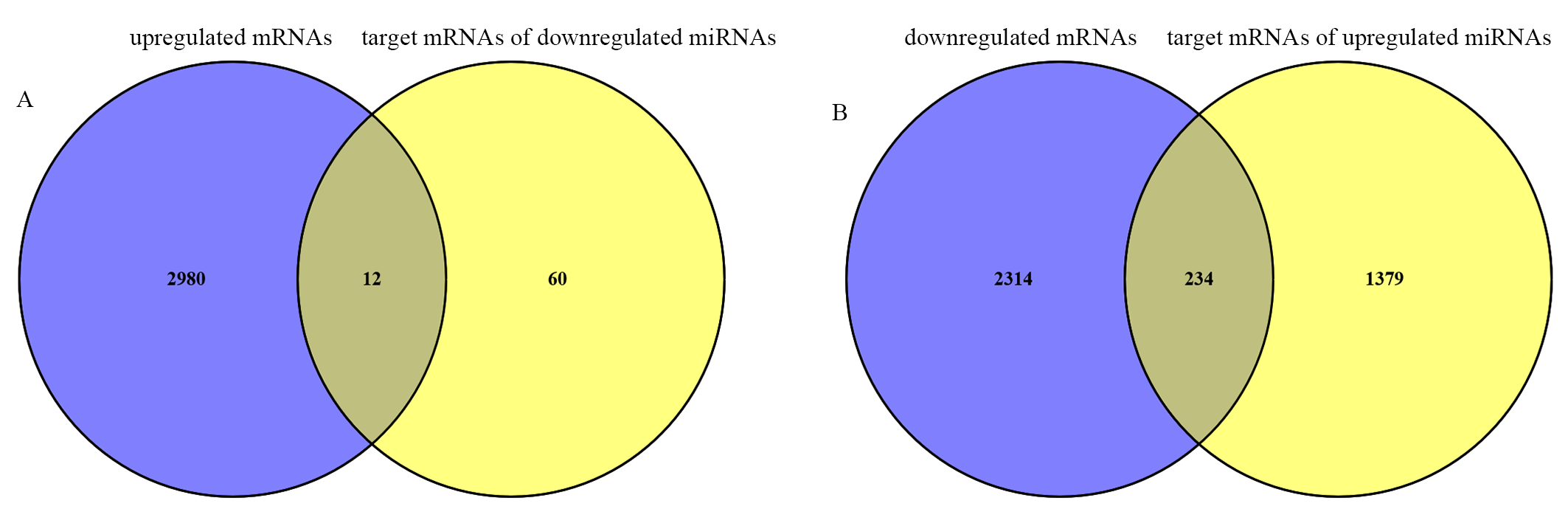

Supplement: Supplemental Figure 2 — The intersection of target mRNAs for miRNA and differentially expressed mRNAs. [file Image_2.TIFF]
